# Trends in Economic Insecurity among Older Adults in California, 2015 - 2023: Using the California Elder Index

**DOI:** 10.1093/geroni/igaf122.3679

**Published:** 2025-12-31

**Authors:** Qianyun Wang, Lauren Vuong, Natalie Kim, Mohammad Khorgamphar, Sithara Diunugala, Shreya Paul, Kathryn Kietzman, D Imelda Padilla-Frausto

**Affiliations:** University of California, Los Angeles, Los Angeles, California, United States; Stanford University, Stanford, California, United States; University of California, Los Angeles, Los Angeles, California, United States; University of California, Los Angeles, Los Angeles, California, United States; UC Irvine Joe C. Wen School of Population & Public Health, Irvine, California, United States; University of California, Los Angeles, Los Angeles, California, United States; UCLA Fielding School of Public Health, Department of Community Health Sciences, Los Angeles, California, United States; UCLA Center for Health Policy Research, Los Angeles, California, United States

## Abstract

The California Elder Index (CEI) provides a better measurement of the income older adults need to cover basic living expenses compared to the Federal Poverty Level (FPL) guidelines. The CEI includes costs for housing, healthcare, food, transportation, and miscellaneous, while the FPL includes food costs only. Using the CEI, we document economic insecurity among older Californians from 2015 to 2023. Since the FPL is widely used to determine eligibility for public programs, we provide CEI and FPL comparisons to show that many older adults with income above the FPL, but below the CEI, lack the income needed for day-to-day expenses. This group falls within the “CEI Income Gap” which we disaggregated into two categories: “CEI Low-Income Gap”(below the CEI and 100-199% FPL) and “CEI Middle-Income Gap” (below the CEI and ≥ 200% FPL). Analyses also include those classified as “very-low-income” (below the CEI and ≤99% FPL). Our findings document trends and persistent disparities in economic insecurity: 1) The percentage of older adults with income below the CEI and FPL steadily increased from 2015 to 2023. 2) Economic insecurity among older adults has shifted, with fewer falling into the CEI Low-Income Gap, and more falling into very-low-income or the CEI Middle-Income Gap. 3) Single older adults living alone consistently face higher rates of economic insecurity than older couples, especially single renters, women, Latinos, and Blacks/African Americans. Our findings underscore the value of using the CEI and an income gap framework to better assess needs and allocate resources for California’s aging population.

